# Balance training benefits chronic ankle instability with generalized joint hypermobility: a prospective cohort study

**DOI:** 10.1186/s12891-023-06179-2

**Published:** 2023-01-27

**Authors:** Zong-chen Hou, Ying-fang Ao, Yue-lin Hu, Chen Jiao, Qin-wei Guo, Nan Li, Yan-fang Jiang, Dong Jiang

**Affiliations:** 1 Department of Sports Medicine of Peking University Third Hospital, Institute of Sports Medicine of Peking University, Beijing Key Laboratory of Sports Injuries, Engineering Research Center of Sports Trauma Treatment Technology and Devices, Ministry of Education, No.49 North Garden Road, 100191 Beijing, China; 2 Research Center of Clinical Epidemiology, Peking University Third Hospital, No.49 North Garden Road, Beijing, 100191 Haidian China

**Keywords:** Chronic ankle instability, Generalized joint hypermobility, Balance training, Muscle strength

## Abstract

**Background:**

Balance training is the first choice of treatment for chronic ankle instability (CAI). However, there is a lack of research on the effects of balance training in CAI with generalized joint hypermobility (GJH). This study is to compare the outcomes of balance training in CAI patients with and without GJH.

**Methods:**

Forty CAI patients were assigned into the GJH group (Beighton ≥ 4, 20) and non-GJH group (Beighton < 4, 20) and they received same 3-month supervised balance training. Repeated measure ANOVA and independent t test were used to analyze self-reported questionnaires (Foot and ankle ability measure, FAAM), the number of patients experiencing ankle sprain, isokinetic muscle strength and postural control tests (Star excursion balance test, SEBT and Balance errors system, BES) before training, post-training immediately, and post-training 3 months, respectively.

**Results:**

At baseline, no differences were found between groups with except for GJH group having poorer SEBT in the posteromedial direction (83.6 ± 10.1 vs 92.8 ± 12.3, %) and in the posterolateral direction (84.7 ± 11.7 vs 95.7 ± 8.7, %). Following the balance training, GJH group demonstrated lower re-sprain ratio (immediately after training, 11.1% vs 23.5%, 3 month after training, 16.7% vs 29.4%) than non-GJH group, as well as greater FAAM-S score, plantarflexion strength and dorsiflexion strength at post-training immediately and 3 months, and both groups improved similarly in the FAAM-A score, muscle strength and balance control (SEBT in the posterior-lateral and posterior-medial directions, and BES scores) compared with baseline.

**Conclusions:**

CAI patients with GJH gained equally even better postural stability and muscle strength after the balance training than the non-GJH patients. Balance training could still be an effective treatment for CAI patients with GJH before considering surgery.

**Trial registration:**

ChiCTR1900023999, June 21^st^, 2019.

**Supplementary Information:**

The online version contains supplementary material available at 10.1186/s12891-023-06179-2.

## Introduction

Ankle sprain is a common injury in sports [1] and 20–40% patients develop into chronic ankle instability (CAI) after the initial sprain, which is often characterized by recurrent ankle sprains [2], decreased postural control and muscle strength [3]. Traditional conservative interventions include balance training, resistance training, joint mobilization, soft tissue mobilization, passive calf stretching and orthotics [4]. Among of them, balance training provided the most consistent improvements in self-reported function for patients [4]. Besides, several systematic reviews [5–7] also summarized the evidence for the efficacy of balance training in enhancing sensorimotor deficits (static and dynamic postural stability, muscle strength, and injury recurrence rates) in subjects with chronic ankle instability. However, there is still a concern about the clinical outcomes of balance training for CAI patients with generalized joint hypermobility (GJH).

Generalized joint hypermobility (GJH) is characterized by increased movement in multiple joints beyond normal ranges expected in a given population [8]. Previous studies inferred that GJH individuals had a higher risk of re-sprain and persistence of complaints [9, 10]. For patients with GJH, increased susceptibility of re-sprain might lead to incomplete or ineffective rehabilitation training, exacerbating outcomes. Therefore, although previous studies [11, 12] established the effectiveness of conservative treatment in alleviating pain in patients with GJH, surgery such as ligament repair or reconstruction are more likely to be recommended in practice for CAI patients with GJH due to the potential of recurrent ligament injuries [10]. By now, there was a lack of prospective study comparing the effects of balance training in CAI cases with GJH and those without, especially for the sprain recurrence, postural control and muscle strength. Whether balance training can have similar good rehabilitation effect for CAI patients with GJH to those without GJH is still unknown.

In the present study, patients with and without GJH with chronic lateral ankle ligament injury were enrolled and underwent same supervised balance training program. Clinical outcomes (subjective patient-reported outcomes, sprain recurrence, postural control, and muscle strength) were analyzed and compared between groups at the post-training immediately and 3 months post-training. We hypothesized that the GJH group would show inferior clinical outcomes after balance training protocols in terms of subjective patient-reported outcomes, sprain recurrence, postural control, and muscle strength. The results would help clinicians and physical therapists to choose therapeutic strategy for the CAI patients with GJH.

## Materials and methods

All methods were performed in accordance with the relevant guidelines and regulations (for example- Declarations of Helsinki) and this is a prospective cohort study and followed STROBE cohort reporting guidelines [13]. From Sep 2018 to May 2020, 40 CAI patients who met the inclusion criteria were screened for enrollment in the study. A priori power analysis was completed using data from a previous study in which the researchers examined the effects of a similar balance-training program [14]. The study was approved by the IRB Medical Committee of the hospital (IRB00006761-M2019164) and the written informed content was obtained from all patients.

### Patient enrollment

The inclusion criteria were (i) age from 18 to 40 years, (ii) a history of at least one episode of lateral ankle sprain (at least 3 months prior to study enrolment) that caused inflammatory symptoms and disrupted activity for at least one day, (iii) reports of joint “giving way” and/or recurrent sprain and/or “feelings of instability” (iv) scoring < 24 on the Cumberland Ankle Instability Tool (CAIT) [15]; (v)grade III [16] injury of anterior talofibular ligament and/or calcaneofibular ligament confirmed by MRI findings, positive anterior drawer test (increased translation of 3 mm compared to the uninjured side or an absolute value of 10 mm of displacement) [17], and positive talar tilt test(10° of absolute talar tilt or 5° difference compared to the contralateral side) by TELOS SD 900 Stress Device (Austin & Associates, inc. USA). Specifically, patients with Beighton score ≥ 4 were enrolled into the GJH group and others were enrolled in the non-GJH group [18]. Patients with combined intra-articular lesions (Osteochondral lesions, osteophyte, impingement, loose body, etc.), history of surgery or neurological disease, and/or acute injury to the lower limb were excluded.

**Fig. 1:**
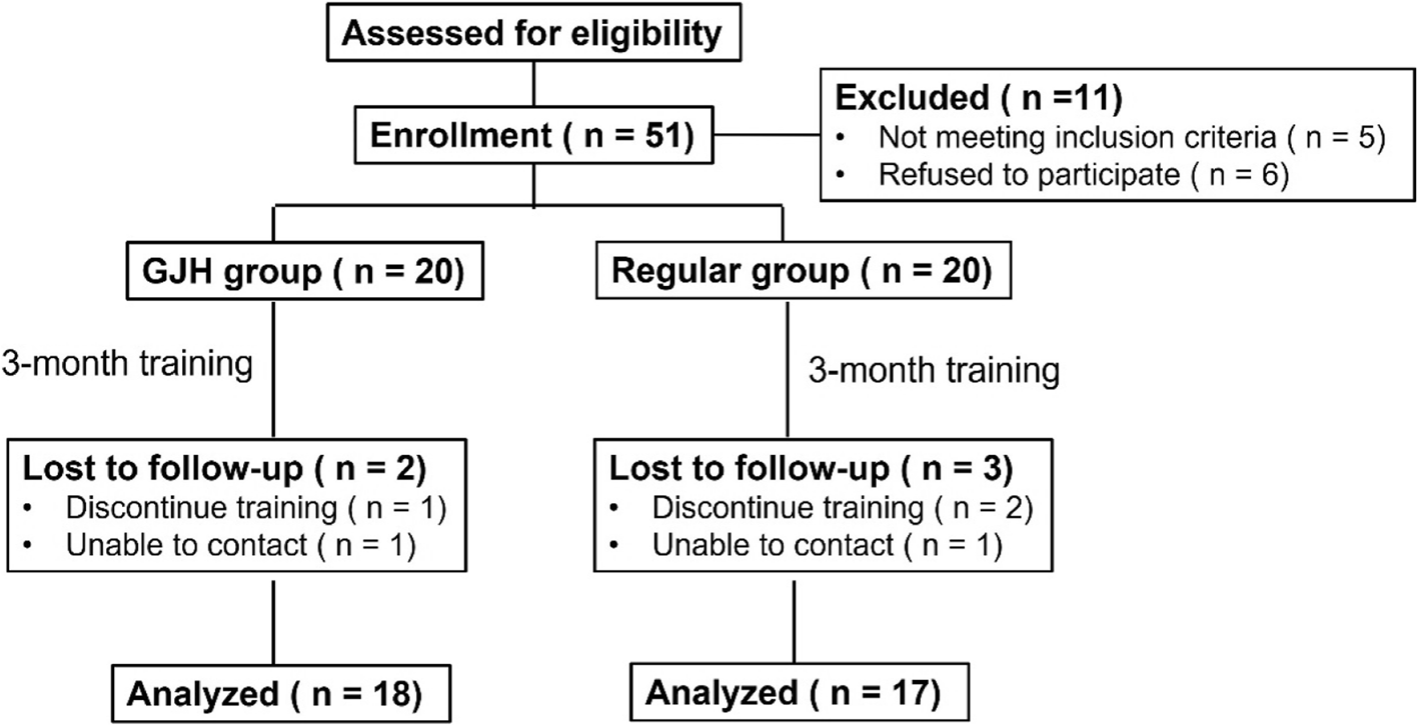
CONSORT flow diagram

Upon enrollment, all the patients’ basic information was collected and evaluated, including the gender, age, height, weight, involved side, interval from first episode of ankle sprain or instability to enrollment, episodes of sprain and Beighton score. A flow diagram based on the CONSORT statement shows the inclusion and exclusion of subjects through the entire study (Fig. 1). Then, all the participants underwent the 12-week balance training intervention by another researcher. The balance training protocol is shown in the Additional file 1: Appendix A. Pre-intervention data-collection session started within 48 h before the intervention and follow-up sessions were performed post-training and 3 months post-training since the pre-intervention data-collection session. The postintervention data-collection session occurred within 48 h after the intervention. Participants were instructed to cease all supervised interventions during the follow-up session. During each data-collection session, we administered the patient-oriented outcomes (Foot and Ankle Ability Measure (FAAM), re-sprain ratio (the number of participants with sprains that caused inflammatory symptoms and disrupted activity for at least one day during follow-up divided by the number of participants recruited) before evaluating the disease-oriented outcomes (isometric ankle strength, postural control).

### Balance training protocol

As was shown in Additional file 1: Appendix A, the balance training protocol was designed based on the widely used protocol from the published papers [19–21]. The protocol includes single-legged stance, wobble board, resistant band and hop exercises. The progressive balance-training program were divided into 24 supervised training sessions, two sessions (60 min each session) per week. The balance training was conducted in the clinics of the hospital and supervised by ZC H.

### Balance testing

Star Excursion Balance Test: Star Excursion Balance Test (SEBT) was used as a measure of dynamic balance. The 3 SEBT directions that we measured were anterior, posterolateral, and posteromedial, as identified for CAI patients in previous research [22]. Before the SEBT, participants were instructed on proper reaching technique and were allowed four practice trials in each direction [23]. They performed 3 consecutive test trials in each direction. The order of directions was randomized. Each participant stood barefoot with the great toe at the center of the SEBT grid. While standing on the involved limb, they reached as far as possible with the non-stance limb along the reach direction. Keeping their hands on their hips, participants lightly touched the line with the most distal portion of the reaching foot and returned to a bilateral stance. The distance was measured from the center of the grid to the farthest reach point. An unsuccessful trial was defined as a trial in which participants lifted their hands off their hips, moved or lifted the stance foot, lifted the heel, transferred weight to the reach foot when touching the measuring tape, did not touch the tape, did not return the reach foot to the starting position, lost their balance, or were unable to maintain a unilateral stance during the trial. Unsuccessful trials were discarded and reattempted. The maximum distance (centimeters) for each reach direction was recorded. Reach distances were normalized to limb length, which was measured from the anterior–superior iliac spine to the distal tip of the medial malleolus.

Balance Error Scoring System: Balance Error Scoring System (BES) is a measure of static balance and consists of 3 stances: double-legged stance, single-legged stance, and tandem stance in a heel-to-toe fashion [24]. Participants performed all stances on firm and foam surfaces (model Balanced; Airex AG, Sins, Switzerland) with their hands on their hips and eyes closed [24]. They performed one practice trial for each condition to ensure proper technique, followed by one test trial. Total errors were counted for each 20-s trial. An error was defined as lifting the hands off the iliac crests; opening the eyes; stepping, stumbling, or falling; moving the hip into more than 30° of abduction; lifting the forefoot or heel; or remaining out of test position for more than 5 s [24]. The maximal possible score for each stance was 10. The total score was used for statistical analysis.

### Isokinetic strength measurement

As described in TW Kaminski’s research [25], isokinetic strength was assessed with a Biodex isokinetic dynamometer (Biodex Medical Systems Inc, Shirley, NY). Each subject’s foot was securely fastened on the chair, with the hip angle 80◦ flexion (0◦ neutral position) and 20°to 30° of knee flexion. Each subject was allowed three submaximal (50% capacity) warm-up repetitions at each velocity to become familiar with the isokinetic test procedure, then performed three maximal concentric test repetitions at 60 and 120°/s on both ankles. The resting interval was approximately one minute between tests for each motion, velocity, and side. At the end of testing, peak torque data was extracted from the torque curves.

### Data analysis

The self-reported function (FAAM and ankle sprain recurrence), balance measures (SEBT test and BES test) and isokinetic muscle strength were analyzed separately at pre-training, post-training, and 6 months. Shapiro–Wilk test was used to assess Normality of data. Independent t test was used to compare the baseline between groups. Two-way repeated measure ANOVA was used to analyze time differences and group differences. Alpha level was set a priori at *P* < 0.05. An a priori power analysis was completed using data from a previous study [20] in which the researchers examined the effects of a similar balance-training program. Based on an α level of 0.05, a power of 0.95, and an effect size of 0.91 determined by the FAAM-Sport, 16 participants were needed. Considering that the settled margin of non-inferiority was 1 point (a delta of 10 points in the FAAM [26]) and the estimated dropout rate was 20%, we enrolled 20 participants in each group. Patients completed at least 21 sessions were included in the analysis and if they did not complete any follow-up measurements, their previous data would also be taken into analysis.

## Results

A total of 40 patients were recruited in the study and 18 patients from GJH group (follow-up rate, 90%) and 17 patients from the non-GJH group (follow-up rate, 85%) completed the final follow-up. 5 patients totally were lost to follow up due to not willing to attend training (GJH group, 1; non-GJH group, 2) or unable to contact during follow-up (GJH group, 1; non-GJH group, 1). As shown in Table 1, there was no significant difference in demographic data of participants, except for the Beighton scores (*P* = 0.013).

**Table 1:** Demographic data of baseline

	GJH group (*N* = 18)	Non-GJH group (*N* = 17)	*P* Value
Sex			.164
Male	8 (44.4%)	7 (41.2%)	
Female	10 (55.6%)	10 (58.8%)	
Age, year	30.2 ± 3.3	29.0 ± 3.3	.894
BMI, kg/m^2^	22.6 ± 3.1	22.5 ± 4.5	.312
CAIT score	14.2 ± 4.4	15.1 ± 6.1	.439
No. of sprains, times	4.4 ± 3.3	5.1 ± 2.9	.664
Duration since last sprain, Months	14.8 ± 6.5	13.6 ± 5.5	.543
Beighton score	6.5 ± 2.2	2.2 ± 1.3	.013*

^*^Means *P* < 0.05

Table 2 exhibits subjective-reported outcomes in both groups. No differences were found in the FAAM-A, FAAM-S, or re-sprain ratio between the GJH group and non-GJH group before training (*P* > 0.05). There were significant time x group interactions with FAAM-S (*P* = 0.021) and re-sprain ratio (*P* = 0.021). GJH group showed significant greater improvements in and FAAM-S and re-sprain ratio than non-GJH group at the post-training and 3 months post-training. In addition, only significant time effect (*P* = 0.012) but not group effect was found in the FAAM-A score, which indicated both groups had similar improvement in the FAAM-A score.

**Table 2:** Subjective-reported outcomes between groups

		Pre-training	Post-training	Post-training 3 months	*P* (Interaction)	*P* (Time)	*P* (Group)
FAAM-A, %	GJH	64.0 ± 6.6	84.3 ± 7.9	92.6 ± 10.5	.882	.012*	.442
	Non-GJH	63.1 ± 7.2	82.5 ± 9.2	90.5 ± 10.5			
FAAM-S, %	GJH	61.5 ± 9.9	93.0 ± 9.4	94.3 ± 9.9	.021*	.022*	.042*
	Non-GJH	63.8 ± 8.2	83.9 ± 9.9	82.7 ± 10.2			
Re-sprain ratio, %	GJH	100 (20/20)	11.1(2/18)	16.7 (3/18)	.039*	< 0.001*	0.015*
	Non-GJH	100 (20/20)	23.5 (4/17)	29.4 (5/17)			

^*^Means *P* < 0.05

Supplementary Table 1 presented the balance changes after training between groups. Before training, GJH group exhibited significant lower SEBT posterolateral (*P* = 0.029) and SEBT posteromedial (*P* = 0.018) than non-GJH group. Significant main time effect was found for SEBT anterior (*P* = 0.012), SEBT posterolateral (*P* = 0.013), SEBT posteromedial (*P* = 0.039), and BES total(*P* = 0.022). After training, both groups showed similarly increase in SEBT posterolateral, SEBT posteromedial, BES total scores at the post-training immediately and post-training 3 months and SEBT anterior at post-training 3 months.

As shown in Supplementary Table 2, no differences were observed at baseline between groups. However, significant group x time interactions were found in the 120°/s dorsiflexion (*P* = 0.012), 60°/s dorsiflexion (*P* = 0.016) and 60°/s plantarflexion strength (*P* = 0.036). Compared with the baseline, GJH group showed consistently increased 60°/s dorsiflexion strength(*P* < 0.05) after balance training while non-GJH group temporarily increased then returned to the baseline(P > 0.05). In addition, the GJH group had a significant increase in the 120°/s dorsiflexion strength at the post-training 3 months(*P* < 0.05) while the non-GJH group maintained the baseline level all the time. Regarding to the 60°/s plantarflexion strength, GJH group had a greater growth than the non-GJH group in the post-training tests (post training, *P* = 0.022, 3 months post-training, *P* = 0.018). After training, both groups had consistently 120°/s plantarflexion and inversion strength and fluctuated (increased at the post training then decreased at the 3 months post-training) 120°/s eversion strength.

## Discussion

The most important finding of our study was that CAI patients with GJH achieved equivalent or even better outcomes (self-reported outcomes, sprain recurrence, balance control, and muscle strength) than those without GJH after balance training. Balance training is a reliable treatment for CAI patients with GJH and could still be an effective treatment before considering surgery.

Before the intervention, no differences in FAAM-A and FAAM-S scores were found between the GJH group and the non-GJH group. This was consistent with previous studies which revealed that the GJH was not a risk factor for the function ability in sport-active individuals [27, 28]. Additionally, our study revealed that the GJH group had a similarly increase in the FAAM-A score as the non-GJH group after the balance training. Consistently, two systematic reviews [11, 12] also established the effectiveness of therapeutic exercise in alleviating pain for the people with symptomatic joint hypermobility. An interesting result of the present study was that the GJH group had higher FAAM-S score and lower re-sprain ratio at post-training immediately and post-training 3 months than the non-GJH group, which indicated that the GJH population might restore a higher sport function and fewer sprain recurrence in the short term than the normal population after balance training. On the one hand, it might be due to the stronger post-training plantar flexion and dorsiflexion strength in the GJH group than the non-GJH group in the follow-up. The strength improvement in the sagittal plane might provide a coordinated effect on the function of the ankle joint [29]. On the other hand, it could be inferred that GJH patients has been experiencing more excessive joint movement due to joint laxity [28] before training so that they might be more sensitive to the balance training, which needs to be examined and explored in the future. These results indicated that the GJH might not a contraindication for the conservative rehabilitation as the previous hypothesis.

Regarding to balance control, the GJH group had worse posteromedial and posterolateral postural stability than the normal CAI patients at the baseline while had similar postural stability improvement after the balance training. Our result suggested that balance training promoted medial–lateral postural stability of GJH patients. The balance control deficits at the baseline were also revealed in previous studies [28, 30]. It has been reported that the GJH adolescents had significantly larger center-of-pressure path length than the normal group across sway tests [28]. In addition, Bates, A V et al. [30] compared responses to forward perturbations between people with GJH and people with normal flexibility and the GJH group indicated impaired balance control during these tasks. Furthermore, the effectiveness of balance training was revealed in the previous studies. Sahin [31] et al. stated that an 8-week proprioception exercise program decreased pain and increased functional status of knee joint in benign joint hypermobility syndrome patients. Our study showed postural control improvements in the ankle joint in terms of the static (BES total at post-training immediately and post-training 3 months) and dynamic medial–lateral postural stability (SEBT posterolateral and posteromedial at post-training immediately and SEBT anterior at post-training 3 months) in the GJH group. The results indicated that the neuromuscular control of GJH population was recoverable, and the effect was parallel to the non-GJH patients.

After balance training, increased plantarflexion, dorsiflexion and eversion strength were found in the both groups while GJH group had a larger increase in the 60°/s plantarflexion strength and more stable increase in dorsiflexion strength than non-GJH group, which might lead to the higher sport function at the 6 months. To, M [32] et al. reported that the concentric and eccentric muscle strength in the people with GJH can strengthen at the same rate as other people without GJH. Contrary to the previous studies [33, 34], our study noted no other strength differences between the hypermobile CAI patients and normal CAI patients at the baseline, which might be due to the fact that these studies recruited the hypermobility patients with pain symptom which might causes more deficits in the muscle strength and limitation in the activities and sport performance. Our study revealed the fact that the CAI with GJH gained greater muscle strength than the normal CAI patients.

To our knowledge, our study was the first prospective cohort trial to focus on the sprain recurrence, postural control and muscle strength of balance training in the CAI with GJH patients. Our results showed that the GJH might not a contraindication but still suitable for the conservative rehabilitation for CAI cases. We should give priority to recommending patients to undergo rehabilitation training before considering surgery for those patients. Although the GJH population has certain characteristics (degeneration of tendons and ligaments, difficult to repair after relaxation, etc.), the results of our study indicated that such patients responded well to balance training, indicating that they had no obvious postural control disorder, and might even have a better recovery of postural control, which needs further study its mechanism. In addition, the mechanism by which muscle strength of GJH population can be increased more significantly needs further study.

The current study had several limitations. First, the current study design was a prospective cohort study with relatively sample size (although meeting the requirements of sample size calculation), so it is unlikely to exclude the subjective selection bias due to the group heterogeneity. In the future, a randomized control trial with a larger sample size needs to be taken to verify the current conclusion. Second, we only included the concentric muscle strength in the muscle evaluation procedure while the changes of eccentric contraction strength should also be considered. Last, the follow-up was relatively short, and future studies are necessary for investigating the long-term effects of balance training in CAI patients with GJH.

In conclusion, CAI patients with GJH achieved equally even better clinical outcomes (sprain recurrence, postural stability, and muscle strength) after the balance training than non-GJH patients. Balance training could still be an effective treatment for CAI patients with GJH before considering surgery.

## Supplementary Information

**Additional file 1:**  **Appendix A.** Balance training protocol.

**Additional file 2:**  **Supplementary Table 1.** Comparison of balance between groups.

**Additional file 3:**  **Supplementary Table 2.** Comparison of muscle strength between the two groups.

## Data Availability

The datasets generated and/or analyzed during the current study are not publicly available due some datasets are written in other articles which haven’t published but are available from the corresponding author on reasonable request.

## Abbreviations

CAI: Chronic ankle instability
GJH: Generalized joint hypermobility
CAIT: Cumberland Ankle Instability Tool
BMI: Body mass index
FAAM: Foot and Ankle Ability Measure
SEBT: Star Excursion Balance Test
BES: Balance Error Scoring System
