# Supplementary material for: Balance training benefits chronic ankle instability with generalized joint hypermobility: a prospective cohort study
Source: BMC Musculoskelet Disord. 2023 Jan 27;24:71. doi: 10.1186/s12891-023-06179-2 (PMC9881354; doi:10.1186/s12891-023-06179-2)
Supplement: Supplementary file 1 — Additional file 1: Appendix A. Balance training protocol. [file 12891_2023_6179_MOESM1_ESM.docx]

**Appendix A.** Balance training protocol.

| Exercise | Description | Progression |
| --- | --- | --- |
| Single-legged stance | Performed up to 60 second per repetition for up to 3 repetitions.  Performed with eyes opened and eyes closed | Progressed when participants could complete a 60-s trial without a loss of balance.  Increased no. of repetitions by one  Changed surface from floor to using the Dyna-Disc ^a^. |
| Wobble board | Slowly moved the board in the plantar- flexion/dorsiflexion and inversion/eversion directions without letting the board contact the floor.  Performed up to 10 repetitions in each direction. | Progressed when participant could complete the task without upper extremity support.  Added rotational directions. |
| Steamboats | Tied a 48-in Thera-band around the unstable ankle.  Positioned stance foot 27-in from where Thera-band was tied.  Performed up to 3 sets of 15 repetitions in each direction (hip flexion, extension, abduction, adduction). | Progressed when participants could complete the repetitions without a loss of balance or fatigue.  Increased no. of repetitions from 10 to 15.  Progressed to next level of resistance with the Thera-band. |
| Single-legged hop | Hopped as far as comfortable in the anterior direction.  Performed up to 15 repetitions. | Progressed when participants could perform the task with minimal ankle and hip motion and no loss of balance on landing.  Increased no. of repetitions from 5 to 10 to 15.  Encouraged increased distance to participants’ tolerance.  Progressed to medial, lateral, and posterior directions. |
| Quadrant hop | Hopped in numbered squares clockwise and counterclockwise while maintaining single-legged stance. | Progressed when participants could complete 2 sets of 5 hops without a loss of balance or fatigue  Made unanticipated directional changes where investigator randomly called out numbers. |
| Single-legged ball catch | Performed up to 3 sets of 20 tosses | Progressed when participants could perform 20 tosses without a loss of balance.  Tossed ball outside participants base of support.  Performed during stance on a Dyna-Disc. |
| Toe touch down | Maintained single-legged stance on a step while lowering the unstable ankle in the anterior, posterior, medial, and lateral directions until the foot contacted floor.  Performed up to 3 sets of 10 repetitions. | Progressed when participants could complete all trials without a loss of balance and with good lower extremity alignment (no eversion collapse)  Increased no. of repetitions from 5 to 10.  Increased height of step from 4 in to 12 in in 2-in increments |
| Hop ups and downs | Hop off step and landed in single-legged stance on floor.  Performed up to 3 sets of 10 repetitions. | Progressed when participants could complete all hops without a loss of balance or fatigue.  Increased no. of repetitions from 5 to 10.  Increased height of step from 4 in to 12 in in 2-in increments  Changed direction of hop.  Hopped up onto step. |

^a^ Exertools, Petaluma, CA. ^b^ The Hygenic Corporation, Akron, OH
