# Supplementary material for: Balance training benefits chronic ankle instability with generalized joint hypermobility: a prospective cohort study
Source: BMC Musculoskelet Disord. 2023 Jan 27;24:71. doi: 10.1186/s12891-023-06179-2 (PMC9881354; doi:10.1186/s12891-023-06179-2)
Supplement: Supplementary file 2 — Additional file 2: Supplementary Table 1. Comparison of balance between groups. [file 12891_2023_6179_MOESM2_ESM.docx]

**Supplementary Table 1. Comparison of balance between groups.**

|  |  | Pre-training | Post-training | Post-training 3 months | *P* (Interaction) | *P* (Time) | *P* (Group) |
| --- | --- | --- | --- | --- | --- | --- | --- |
| SEBT, % |  |  |  |  |  |  |  |
| Anterior | GJH | 82.7(6.0) | 84.2(10.7) | 105.7(6.1) | .882 | .012* | .442 |
|  | Non-GJH | 85.3(7.2) | 86.5(9.9) | 103.2(10.5) |  |  |  |
| Posterolateral | GJH | 83.6(10.1) † | 111.7(9.2) | 110.4(6.2) | .592 | .013* | .142 |
|  | Non-GJH | 92.8(12.3) | 105.9.7(8.8) | 116.5(9.9) |  |  |  |
| Posteromedial | GJH | 84.7(11.7) † | 106.1(8.7) | 101.3(7.5) | .661 | .039* | .598 |
|  | Non-GJH | 95.7(8.7) | 109.1(9.9) | 98.9(7.3) |  |  |  |
| BES total | GJH | 19.49(3.32) | 12.77(1.33) | 13.77(2.3) | .319 | .022* | .865 |
|  | Non-GJH | 18.66(2.04) | 11.65(1.77) | 12.81(2.68) |  |  |  |

* Means *P* < .05.

† Means significant group difference at the baseline, *P* < .05.
