# Supplementary material for: Balance training benefits chronic ankle instability with generalized joint hypermobility: a prospective cohort study
Source: BMC Musculoskelet Disord. 2023 Jan 27;24:71. doi: 10.1186/s12891-023-06179-2 (PMC9881354; doi:10.1186/s12891-023-06179-2)
Supplement: Supplementary file 3 — Additional file 3: Supplementary Table 2. Comparison of muscle strength between the two groups. [file 12891_2023_6179_MOESM3_ESM.docx]

**Supplementary Table 2. Comparison of muscle strength between the two groups.**

|  | Group | Pre-training | Post-training | 3 months post-training | *P* (Interaction) | *P* (Time) | *P* (Group) |
| --- | --- | --- | --- | --- | --- | --- | --- |
| 120°/s, N*m/kg |  |  |  |  |  |  |  |
| Plantarflexion | GJH | 33.3(11.2) | 49.5(11.8) | 46.7(9.0) | .437 | <.001* | .368 |
|  | Non-GJH | 33.9(7.0) | 43.3(9.8) | 42.8(8.9) |  |  |  |
| Dorsiflexion | GJH | 16.6(6.5) | 21.4(5.2) | 27.6(7.9) | .012* | .018* | .024* |
|  | Non-GJH | 15.7(5.2) | 20.1(8.5) | 17.8(7.6) |  |  |  |
| Eversion | GJH | 26.7(9.3) | 37.2(6.2) | 26.5(14.4) | .371 | .048* | .614 |
|  | Non-GJH | 29.2(7.6) | 38.5(8.7) | 30.4(11.6) |  |  |  |
| Inversion | GJH | 24.1(7.7) | 33.6(7.1) | 32.4(11.1) | .224 | .014* | .745 |
|  | Non-GJH | 21.2(5.8) | 30.2(8.7) | 30.1(9.0) |  |  |  |
| 60°/s, N*m/kg |  |  |  |  |  |  |  |
| Plantarflexion | GJH | 39.8(18.5) | 63.8(19.6) | 68.6(19.6) | .036* | <.001* | .023* |
|  | Non-GJH | 42.1(10.7) | 55.7(18.6) | 56.2(15.6) |  |  |  |
| Dorsiflexion | GJH | 22.2(7.2) | 27.6(8.6) | 30.3(14.7) | .023* | .028* | .022* |
|  | Non-GJH | 18.6(9.6) | 27.1(11.3) | 19.3(10.7) |  |  |  |
| Eversion | GJH | 30.1(7.1) | 32.1(6.2) | 34.4(11.9) | .891 | .467 | .724 |
|  | Non-GJH | 30.2(10.8) | 30.4(9.3) | 32.6(14.1) |  |  |  |
| Inversion | GJH | 27.1(9.7) | 32.6(6.2) | 34.8(10.9) | .524 | .545 | .935 |
|  | Non-GJH | 29.1(11.4) | 28.9(9.2) | 34.9(11.0) |  |  |  |

* means *P* < .05.
